# The protein kinase Cmk2 negatively regulates the calcium/calcineurin signalling pathway and expression of calcium pump genes *PMR1* and *PMC1* in budding yeast

**DOI:** 10.1186/s12964-019-0320-z

**Published:** 2019-01-21

**Authors:** Huihui Xu, Tianshu Fang, Hongbo Yan, Linghuo Jiang

**Affiliations:** Laboratory for Yeast Molecular and Cell Biology, School of Agricultural Engineering and Food Science, Shandong University of Technology, Zibo, Shandong China

**Keywords:** *Saccharomyces cerevisiae*, Cmk2, Pmr1, Calcineurin, Crz1, Calcium signalling

## Abstract

Through a genome-wide screen we have identified calcium-tolerant deletion mutants for five genes in the budding yeast *Saccharomyces cerevisiae*. In addition to *CNB1* and *RCN1* that are known to play a role in the calcium signalling pathway, the protein kinase gene *CMK2*, the sphingolipid homeostasis-related gene *ORM2* and the gene *SIF2* encoding the WD40 repeat-containing subunit of Set3C histone deacetylase complex are involved in the calcium sensitivity of yeast cells to extracellular calcium. Cmk2 and the transcription factor Crz1 have opposite functions in the response of yeast cells to calcium stress. Deletion of *CMK2* elevates the level of calcium/calcineurin signalling and increases the expression level of *PMR1* and *PMC1*, which is dependent on Crz1. Effects of Cmk2 on calcium sensitivity and calcium/calcineurin signalling are dependent on its kinase activity. Therefore, Cmk2 is a negative feedback controller of the calcium/calcineurin signalling pathway. Furthermore, the *cmk2 crz1* double deletion mutant is more resistant than the *crz1* deletion mutant, suggesting that Cmk2 has an additional Crz1-independent role in promoting calcium tolerance.

**Electronic supplementary material:**

The online version of this article (10.1186/s12964-019-0320-z) contains supplementary material, which is available to authorized users.

## Background

Calcium ions regulate many cellular processes in both prokaryotes and eukaryotes [1–5]. Regulation of intracellular calcium homeostasis and the calcium/calcineurin signalling pathway are highly conserved in eukaryotic cells [6, 7]. In the budding yeast *Saccharomyces cerevisiae*, cytosolic calcium homeostasis is regulated by Ca^2+^ transporters and sequestrators in the plasma and organellar membranes. Transient increases in cytosolic Ca^2+^ activate the calcium/calcineurin signalling pathway. Sustained Ca^2+^ accumulation in the cytosol is prevented by a Ca^2^ sequestration system composed of the calcium pump Pmc1 and the Ca^2+^/H^+^ exchanger Vcx1 in the vacuolar membrane as well as the calcium pump Pmr1 and the Ca^2+^/H^+^ exchanger Gdt1 in the ER/Golgi secretory pathway [8–10]. In our recent studies, we have demonstrated that Rch1 is a novel negative regulator of calcium uptake in the plasma membrane in the budding yeast and the human yeast pathogen *Candida albicans* [11–15].

As a eukaryotic model organism, *S. cerevisiae* has been proven to be a valuable genetic tool to elucidate mechanisms regulating calcium homeostasis and calcium signalling in eukaryotic cells as well as to characterize gene-drug and pathway–drug interactions [16–20]. In our previous study, from all nonessential genes in the genome we have identified 120 genes that are involved in the tolerance of the budding yeast to high levels of extracellular calcium [21]. To explore genes involved in the sensitivity of yeast cells to extracellular calcium, we screened the same diploid deletion mutant set for calcium-tolerant gene deletion mutants. In this study, we have identified a total of five genes whose deletion leads to the tolerance of *S. cerevisiae* cells to high levels of extracellular calcium. Furthermore, we show that the protein kinase Cmk2 is a negative feedback controller of the calcium/calcineurin signalling pathway.

## Methods

### Strains and reagents

The *S. cerevisiae* haploid wild type BY4741, the diploid wild type BY4743 strain and their isogenic deletion mutant sets used in this study were purchased from Invitrogen Inc. [21–23] (Table 1) Yeast cells were grown at 30 °C in YPD medium (1% yeast extract, 2% peptone, 2% glucose) or SD medium (0.67% yeast nitrogen base without amino acids, 2% glucose, and auxotrophic amino acids as needed). Chemicals were purchased from Sangon Biotech (Shanghai, China), except that cyclosporine A (CsA) and O-nitrophenyl-β-D-galactopyranoside (ONPG) were purchased from Sigma (Beijing, China) and the antibiotic nourseothricin was purchased from Golden Biotechnology Inc. (Missouri, USA).

**Table 1:** *S. cerevisiae* strains used in this study

Strain	Genotype	Source
BY4743	*MATa/α his3Δ/ his3Δ1 leu2Δ0/leu2Δ0 met15Δ0/met15Δ0 ura3Δ0/ura3Δ0*	Invitrogen Inc.
	BY4743 *cmk2::kan*MX4/*cmk2::kan*MX4	Invitrogen Inc.
	BY4743 *cnb1::kan*MX4/*cnb1::kan*MX4	Invitrogen Inc.
	BY4743 *orm2::kan*MX4/*orm2::kan*MX4	Invitrogen Inc.
	BY4743 *rcn1::kan*MX4/*rcn1::kan*MX4	Invitrogen Inc.
	BY4743 *sif2::kan*MX4/*sif2::kan*MX4	Invitrogen Inc.
BY4741	*MATa his3Δ1 leu2Δ0 met15Δ0 ura3Δ0*	Invitrogen Inc.
	BY4741 c*mk1::kan*MX4	Invitrogen Inc.
	BY4741 c*mk2::kan*MX4	Invitrogen Inc.
	BY4741 *cnb1::kan*MX4	Invitrogen Inc.
	BY4741 *vcx1::kan*MX4	Invitrogen Inc.
	BY4741 *crz1::kan*MX4	Invitrogen Inc.
	BY4741 *cmk2::nat*MX4	This study
	BY4741 *vcx1::nat*MX4	This study
	BY4741 *cmk2::nat*MX4 *cmk1::kan*MX4	This study
	BY4741 *cmk2::nat*MX4 *cnb1::kan*MX4	This study
	BY4741 *cmk2::nat*MX4 *vcx1::kan*MX4	This study
	BY4741 *cmk2::nat*MX4 *crz1::kan*MX4	This study
	BY4741 *vcx1::nat*MX4 *cnb1::kan*MX4	This study

### Genome-wide screen for calcium-tolerant mutations

For primary screens for calcium-tolerant strains, the collection of homozygous diploid deletion mutants of nonessential 4757 genes was replicated onto YPD plates with 0.4 M CaCl2 and YPD plates without supplemented CaCl_2_ (as controls). respectively. These plates were incubated at 30 °C for 2–3 days. A mutant with a relative colony size increased by more than 30% as compared to the average size of its surrounding mutants on YPD plates containing 0.4 M CaCl_2_ but not on YPD plates without supplemented CaCl_2_, was identified as a potential calcium-tolerant mutant. Primary screen was repeated two times. Tolerant mutants were subjected to a secondary screen by a serial dilution assay method as described [22, 24]. The suppressive effects of cyclosporine A on the phenotypes of calcium-tolerant mutants were examined by supplementing 50 μg/ml CsA into YPD plates with or without CaCl_2_ [25].

### Construction of double-gene deletion mutants

To study the interaction between *CMK2* and genes encoding components of the calcium/calcineurin signalling pathway in calcium sensitivity, we created their double-gene deletion mutants in the BY4741 genetic background. First, *kan*MX4 markers in single-gene deletion mutants for *CMK2* and *VCX1* (*cmk2::kan*MX4 and *vcx1::kan*MX4) were replaced by the *nat*MX4 in the plasmid p4339 to generate *cmk2::nat*MX4 and *vcx1::nat*MX4 strains as described previously [26]. The *cmk2::nat*MX4 cassette was PCR amplified from the genomic DNA of the *cmk2::nat*MX4 strain with a pair of primer CMK2-F (5’ CGATATTGTT CAAGATCAGC AG 3)/CMK2-R (5’ ATGCCATGAA GTGTAGCTGC 3′), which locate 100-bp upstream and downstream, respectively, of the open reading frame (ORF) of the *CMK2* gene, and used for replacing the *CMK2* ORFs in *kan*MX4 mutants for target genes to generate their double-gene deletion mutants. The deletion of *CMK2* gene in these double-gene deletion mutants was confirmed by PCR amplification with detection primer pair CMK2-DF (5’ GAGGCTTATC TTAGAACCC 3′)/CMK2-DR (5’ AGAACCCGTT AGGCAACTAC 3′), which flank the CMK2-F and CMK2-R. Similarly, the double-gene deletion mutant between *CNB1* and *VCX1* was constructed (Table 1).

### Galactosidase activity assay

To measure the calcineurin dependent response element (CDRE)-driven β-galactosidase activity in the wild type and the *cmk2/cmk2* mutant, we integrated the *Stu*I-precut plasmid DNA containing the 4 × CDRE-*lac*Z reporter into the *AUR1* locus of these strains as described [12, 27]. The *PMC1*-*lac*Z reporter and *PMR1*-*lac*Z reporter were described in our previous study [28]. The β-galactosidase activity was determined using the substrate ONPG as described [29–31]. Data are mean ± SD from six independent experiments.

### DNA manipulation

To clone the full-length gene *CMK2* into the centromeric vector pHAC111 [26], a DNA fragment containing the 776-bp promoter, the 1344-bp open reading frame (ORF) and the 341-bp terminator region of *CMK2* was amplified with a pair of primers ScCMK2-clonF (5′ gtatgggtag catgcctgca gGACACAATG ATAGGCACAA CGC 3′; lower-case sequence from the pHAC111 vector) and ScCMK2-clonR (5′ aaaacgacgg ccagtgaatt cTGACATTGA CGTTAGCGAT GACT 3′;; lower-case sequence from pHAC111), and cloned between *Pst*I and *Eco*RI sites in pHAC111 through homologous recombination, which yielded pHAC111-CMK2.

Next, we amplified the DNA fragment CMK2-UP containing the 842-bp promoter and the 225-bp *CMK2* ORF region upstream of the AAG codon (lysine) with primers ScCMK2-mutant-F1 (5’ CCTTTCTAGA GCGTTTTCTG TG 3′) and ScCMK2-mutant-R1 (5’ CAATAAGATG GCTATAGCAA CATCTTCATT TGTGG 3′; underlined is the mutated codon of AAG). Similarly, the DNA fragment CMK2-DOWN containing the 1115-bp *CMK2* ORF region downstream of the AAG codon and the 414-bp terminator was PCR amplified with primers ScCMK2-mutant-F2 (5′ GAAGATGTTG CTATAGCCAT CTTATTGAAG AAGGCATTGC 3′; underlined is the mutated codon of AAG) and ScCMK2-mutant-R2 (5’ CTCAGAGAAG AACCACGGTG 3′). The two fragments CMK2-UP and CMK2-DOWN were fused together by PCR with primers ScCMK2-clonF and ScCMK2-clonR, and the fused product was cloned between *Pst*I and *Eco*RI sites in pHAC111 through homologous recombination, yielding pHAC111-CMK2M, expressing a point (K76A) mutant form of Cmk2 under its own promoter. All constructs were confirmed by DNA sequencing.

### Statistical analysis

Data are presented as means ± SD. Significant differences were analyzed by GraphPad Prism version 4.00 (USA). *P* values of < 0.05 were considered to be significant.

## Results

### Screen for calcium-tolerant gene deletion mutants

**Fig. 1:**
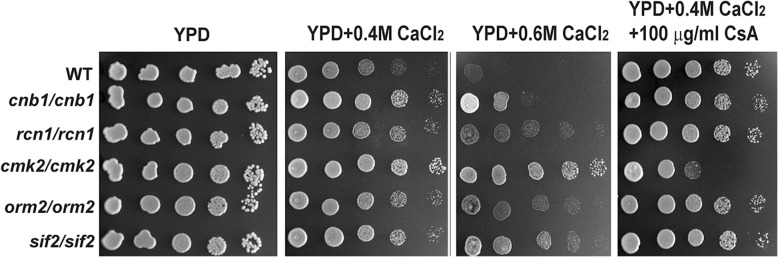
Phenotypes of calcium-tolerant gene deletion mutants. Cells of the wild-type BY4743 and five deletion mutants identified from the library screen were grown at 30 °C in liquid YPD overnight, serially diluted by 10 times and spotted on YPD plates with or without supplemented CaCl_2_ or cyclosporine A (CsA) as indicated, respectively. Plates were incubated for 2 to 3 days at 30 °C

In our previous study [21], through a genome-wide approach we have identified 120 genes that are involved in the tolerance of *S. cerevisiae* cells to high levels of extracellular calcium. To explore genes involved in the sensitivity of yeast cells to extracellular calcium, we screened the same deletion library for calcium-tolerant gene deletion mutants. As a result, we revealed that deletion of *CNB1*, *RCN1*, *CMK2*, *ORM2* or *SIF2* caused yeast cells to be tolerant to 0.4 M CaCl_2_, and these phenotypes were more dramatical in the presence of 0.6 M CaCl_2_ (Fig. 1). The calcium tolerant-phenotypes of yeast cells lacking *RCN1*, *ORM2* or *SIF2* on YPD plate containing 0.4 M CaCl_2_ could be slightly suppressed by the addition of the cyclosporin A (CsA), the specific inhibitor of calcineurin (Fig. 1).

*RCN1* encodes a protein involved in calcineurin regulation during calcium signalling and has similarity to human DSCR1 that is found in the Down Syndrome candidate region [32]. *CNB1* encodes a regulatory subunit of calcineurin, a Ca^2+^/calmodulin-regulated type 2B protein phosphatase, which dephosphorylates the transcription factor Crz1, and promotes its nuclear localization [9]. Our observation that deletion of *CNB1* leads to a calcium-tolerant phenotype in the wild type BY4743 background is consistent with previous studies [33, 34], showing that inactivation of calcineurin including deletion of *CNB1* restores the calcium-tolerance of yeast cells lacking *PMC1*, since calcineurin decreases calcium tolerance of *pmc1* cells by inhibiting the function of the vacuolar H^+^/Ca^2+^ exchanger Vcx1. Therefore, it is not surprising to note that the difference in calcium sensitivity between the wild type and the *cnb1/cnb1* mutant is abolished by CsA, since it suppresses the function of calcineurin in the wild type (Fig. 1). However, interestingly, yeast cells lacking *CMK2* were sensitive to calcium stress in the presence of CsA as compared to the wild type (Fig. 1). This suggests that the calcium tolerance due to deletion of *CMK2* requires the presence of a functional calcineurin.

### Calcium tolerance of *cmk2* cells requires the calcium/calcineurin signalling in response to high levels of extracellular calcium

**Fig. 2:**
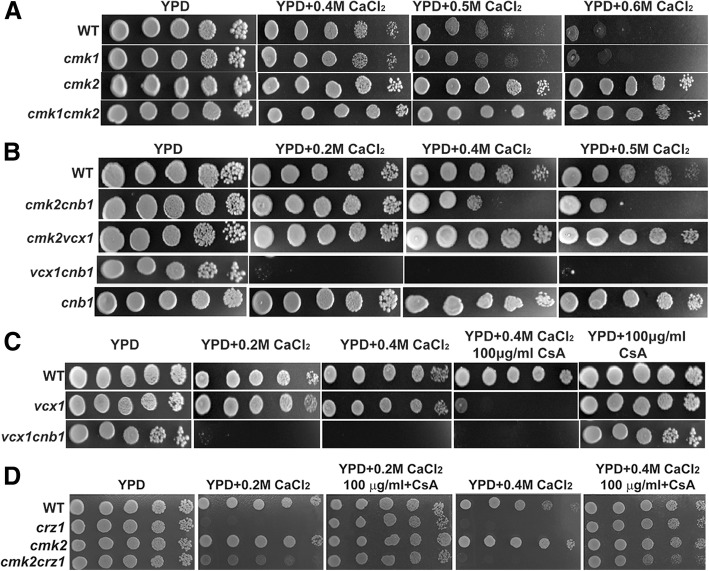
Phenotypes of the wild type BY4741, the single-gene deletion mutant for *CMK2* and double-gene deletion mutants between *CMK2* and one of other genes indicated. Strains were grown at 30 °C in liquid YPD overnight, serially diluted by 10 times and spotted on YPD plates with or without reagents indicated. Plates were incubated for 2 to 3 days at 30 °C

As homologs of mammalian Cam Kinase II, Cmk2 and its paralog Cmk1 are calmodulin-dependent protein kinases (CaMK) that play a role in stress response in *S. cerevisiae* [35, 36]. The yeast CaMK, encoded by both *CMK1* and *CMK2*, and the calcineurin act independently in promoting survival of pheromone-induced growth arrest [35]. However, deletion of *CMK1* did not lead to a calcium-tolerant phenotype of the wild type BY4743 cells as deletion of *CMK2*, neither did affect the calcium-tolerant phenotype of yeast cells lacking *CMK2* (Fig. 2a). This indicates only Cmk2, but not Cmk1, is involved in the response of yeast cells to high levels of external calcium. To examine if the calcium-tolerant phenotype of yeast cells lacking *CMK2* is related to the calcium/calcineurin signalling pathway, we constructed double-gene deletion mutants between *CMK2* and genes encoding various components of the calcium/calcineurin signalling pathway.

Although deletion of *CNB1* causes yeast cells to be calcium-tolerant in the haploid BY4741 background as in the diploid BY4743 background, further deletion of *CNB1* led to a calcium-sensitive phenotype for yeast cells lacking *CMK2* (Fig. 2b). In contrast, deletion of *VCX1* did not have an impact on the calcium sensitivity of the wild type BY4741 cells harboring a functional calcineurin, and neither did on the calcium-tolerant phenotype for yeast cells lacking *CMK2* (Fig. 2b and c). Like in yeast cells lacking *CMK2*, further deletion of *CNB1* also led to a calcium-sensitive phenotype for yeast cells lacking *VCX1* (Fig. 2c). As expected [10], deletion of *CRZ1* alone led to a hypersensitivity of BY4741 cells to calcium stress (Fig. 2b). Yeast cells lacking both *CRZ1* and *CMK2* showed a hypersensitive phenotype on YPD plate containing 0.4 M CaCl_2_ with a similar degree to cells lacking *CRZ1* alone. In contrast, yeast cells lacking both *CRZ1* and *CMK2* showed a sensitive phenotype on YPD plate containing 0.2 M CaCl_2_ in a less degree than cells lacking *CRZ1* alone (Fig. 2d). However, all these phenotypes could be suppressed by the specific inhibitor of calcineurin, CsA (Fig. 2d). This indicates that Cmk2 and Crz1 have independent and opposite functions in the calcium sensitivity of yeast cells. Taken together, our data suggest that the calcium tolerance due to deletion of *CMK2* is dependent on Crz1 under high concentrations of calcium, but is independent of Crz1 under lower concentrations of calcium.

### Deletion of *CMK2* leads to increased expression of *PMR1* and *PMC1* due to activation of the calcium/calcineurin signalling

**Fig. 3:**
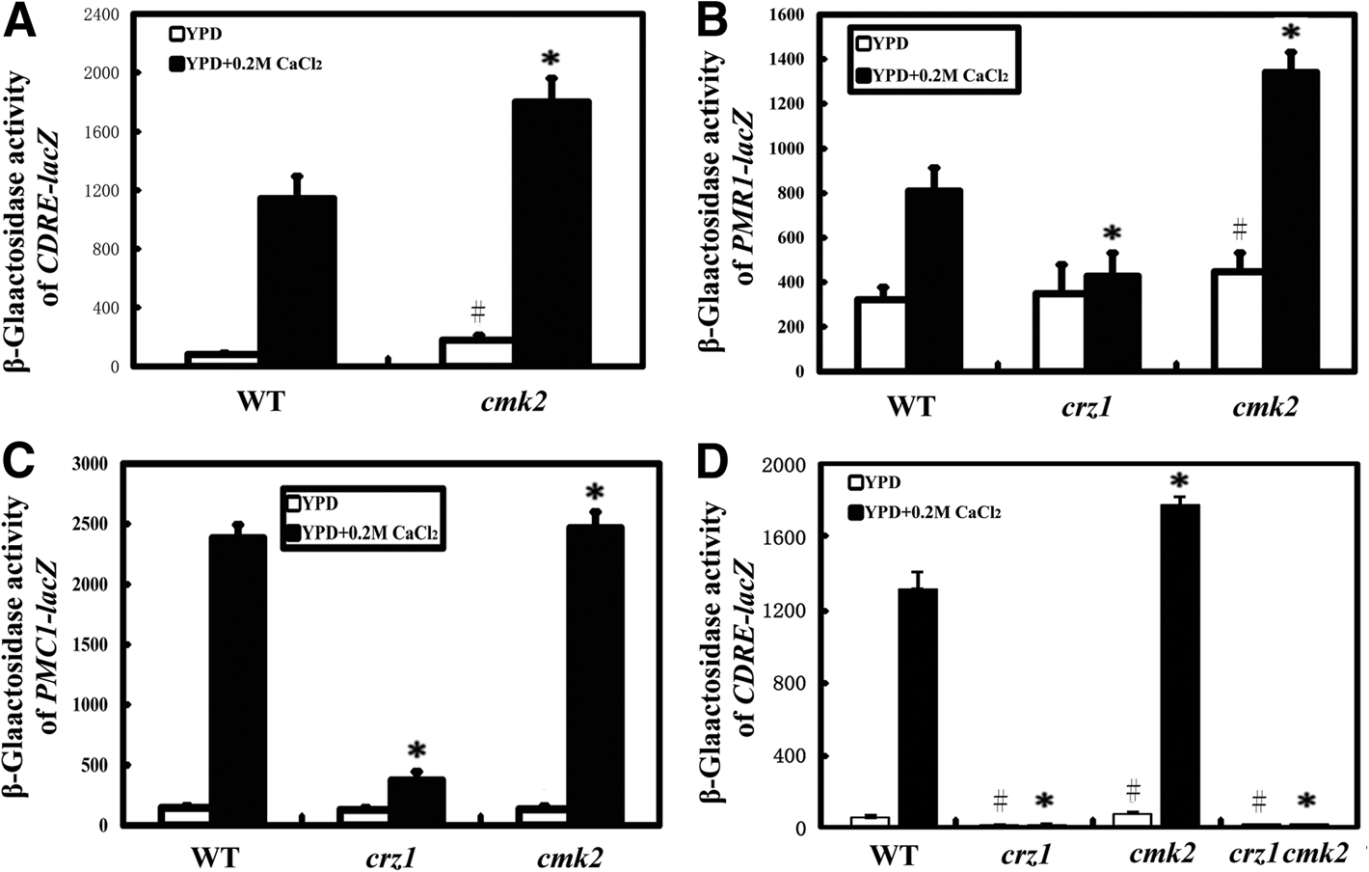
Deletion of *CMK2* increases the activation level of calcium/calcineurin signaling and the expression level of *PMR1*. Galactosidase activities of 4xCDRE-*lac*Z (**a** and **d**), *PMR1*-*lac*Z (**b**) or *PMC1*-*lac*Z (**c**) in the wild-type (WT), the *cmk2* mutant and the *crz1* mutant growing in log phase. Symbols # and * show statistically significant differences (*P* < 0.05) between the wild type and each of the two mutants in the absence or presence of 0.2 M CaCl_2_, respectively. Values were means of six independent experiments

Next, we examined if deletion of *CMK2* would activate the calcium/calcineurin signalling. We examined the calcium-dependent response element (CDRE)-*lac*Z activity in the wild type and the *cmk2* mutant. As compared to the wild type, the CDRE-*lac*Z activity in the *cmk2* mutant was significantly increased in the absence or presence of 0.2 M CaCl_2_ (Fig. 3a). This suggests that deletion of *CMK2* elevates the activation levels of the calcium/calcineurin signaling independent of calcium stress.

Activation of calcium/calcineurin signalling induces the expression of *PMR1* and *PMC1*, but not *VCX1* [21, 34, 37]. Next, we examined the expression of *PMR1* and *PMC1* using their *lac*Z reporters in the wild type, the *crz1* mutant and the *cmk2* mutant. The *lac*Z activities of *PMR1* and *PMC1* were induced in the wild type in response to 0.2 M CaCl_2_ (Fig. 3b and c). This is consistent with the previous observation [34]. In the absence of 0.2 M CaCl_2_, there was no significant difference in the *lac*Z activities of *PMR1* or *PMC1* between the wild type and *the crz1* mutant, but there was a significant increase in the *lac*Z activities of *PMR1* and *PMC1* in *the cmk2* mutant as compared to the wild type (Fig. 3b and c).

In the presence of 0.2 M CaCl_2_, there was a significant decrease in the *lac*Z activities of both *PMR1* or *PMC1* in *the crz1* mutant as compared to the wild type (Fig. 3b and c). In contrast, there was a significant increase in the *lac*Z activity of both *PMR1* and *PMC1*, in the *cmk2* mutant as compared to the wild type (Fig. 3b and c). Therefore, deletion of *CMK2* increase the expression level of both *PMR1* and *PMC1*.

### Enhanced CDRE-*lac*Z activity due to deletion of *CMK2* is dependent on Crz1

As we described above, expression of both CDRE-*lac*Z and *PMR1*-*lac*Z reporters was increased in the absence of added calcium in the *cmk2* mutant as compared to the wild type (Fig. 3a and b). To examine if this effect is due to enhanced Crz1 activity, we measured the activity of CDRE-*lac*Z in the *crz1*, the *cmk2* and the *crz1/cmk2* mutants. As expected, in the absence or presence of 0.2 M CaCl_2_, the activity of CDRE-*lac*Z was almost abolished in both the *crz1* mutant and the *crz1/cmk2* mutant, albeit increased in the *cmk2* mutant (Fig. 3d). In addition, the activity of CDRE-*lac*Z was significantly lower in either the *crz1* mutant or the *crz1/cmk2* mutant than the wild type strain in the absence of added calcium, although no significant difference in the activity of CDRE-*lac*Z was observed between the *crz1* mutant and the *crz1/cmk2* mutant in the absence or presence of 0.2 M CaCl_2_ (Fig. 3d). Taken together, these data suggest that the enhanced CDRE-*lac*Z activity in the *cmk2* mutant was dependent on Crz1 in the absence or presence of calcium stress and that a basal level of Crz1 activity is present in yeast cells growing in YPD medium.

### Effect of Cmk2 on the CDRE-*lac*Z activity is dependent on its catalytic activity

**Fig. 4:**
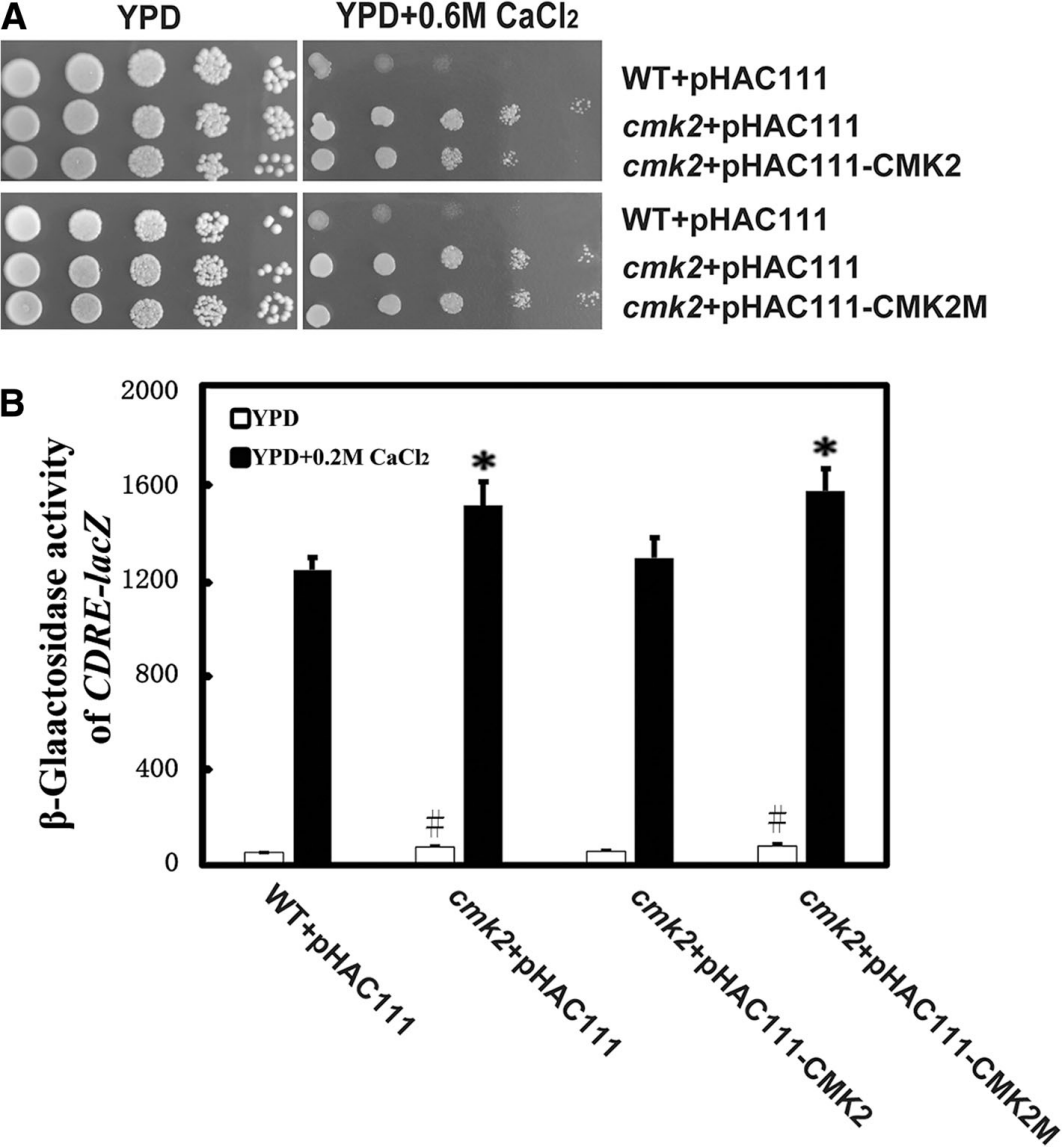
Effects of Cmk2 on both calcium sensitivity and CDRE-*lac*Z activity are dependent on its catalytic activity. (**a**), cells of the wild-type BY4741 and the *cmk2* mutant carrying the pHAC111 vector, pHAC111-CMK2 or pHAC111-CMK2M were grown at 30 °C in liquid SD-LEU medium overnight, serially diluted by 10 times and spotted on YPD plates with or without 0.6 M CaCl_2_, respectively. Plates were incubated for 2 to 3 days at 30 °C. (**b**), Galactosidase activities of 4xCDRE-*lac*Z in the wild-type (WT) BY4741and the *cmk2* mutant, carrying the pHAC111 vector, pHAC111-CMK2 or pHAC111-CMK2M as indicated. They were grown at 30 °C in liquid SD-LEU medium overnight before they were inoculated to, and grown in, YPD medium to log phase. Cells were further grown in the absence or presence of 0.2 M CaCl_2_ for 2 h before they were collected for protein extraction and *lac*Z activity assay. Symbols # and * show statistically significant differences (*P* < 0.05) between the wild type and each of other strains in the absence or presence of 0.2 M CaCl_2_, respectively. Values were means of six independent experiments

We examined if the catalytic activity of the protein kinase Cmk2 is responsible for its suppressive effect on the CDRE-*lac*Z activity. The critical lysine residue in ScCmk2 was identified from the amino acid alignment between *S. cerevisiae* ScCmk2, *Schizosaccharomyces pombe* SpCmk2 [38] and *S. cerevisiae* ScRck2 [26, 39] (Additional file 1: Figure S1). We first constructed the plasmids pHAC111-CMK2 and pHAC111-CMK2M, expressing the wild type Cmk2 and its kinase-dead point mutant (K76A) variant of Cmk2. Introduction of pHAC111-CMK2, but not pHAC111-CMK2M, partially suppressed the calcium-tolerant phenotype of the *cmk2* mutant (Fig. 4a). This suggests that the catalytic activity of Cmk2 is required for the function of Cmk2 in the sensitivity of yeast cells to high levels of extracellular calcium stress.

Next, we introduced the vector pHAC111 to the wild type BY4741 with the CDRE-*lac*Z reporter and the *cmk2* mutant with the CDRE-*lac*Z reporter, respectively. In addition, pHAC11-CMK2 and pHAC11-CMK2M were introduced into the *cmk2* mutant strain with the CDRE-*lac*Z reporter, respectively. As expected, in the absence or presence of 0.2 M CaCl_2_, the *cmk2* mutant carrying the pHAC111 vector showed a higher CDRE-*lac*Z activity than the wild type carrying pHAC111 (Fig. 4b). Introduction of pHAC11-CMK2, but not pHAC11-CMK2M, suppressed the enhanced effect of *cmk2* deletion on the CDRE-*lac*Z activity in the absence or presence of 0.2 M CaCl_2_ (Fig. 4b). This indicates that the negative effect of Cmk2 on the CDRE-*lac*Z activity is dependent on its kinase activity.

## Discussion

Through a chemical genetic screen, we have identified five genes, *CNB1*, *RCN1*, *ORM2*, *SIF2* and *CMK2*, that are involved in the sensitivity of *S. cerevisiae* cells to high levels of extracellular calcium. Two genes, both *CNB1* and *RCN1*, encode regulators of calcineurin. Calcineurin not only induces the expression of *PMC1* and *PMR1* through the action of the transcription factor Crz1, but also inhibits in a Crz1-independent fashion the activity of Vcx1, the high capacity, low affinity vacuolar Ca^2+^ exchanger [37, 40]. The calcium-tolerance of yeast cells lacking *CNB1* is likely due to the release of the inhibitory effect of calcineurin on Vcx1 activity, which is supported by our observation that further deletion of *VCX1* reversed the phenotype of cells lacking *CNB1* to be hypersensitive to calcium stress with a similar degree to that of cells lacking *VCX1* in the presence of CsA (Fig. 2c).

*ORM2* encodes a regulator of the sphingolipid homeostasis. Our finding is consistent with previous observations that sphingolipids play roles in calcium homeostasis, protein trafficking/exocytosis, longevity and cellular aging, nutrient uptake, and the interaction of sphingolipids and antifungal drugs in *S. cerevisiae* [41]. In mammalian cells, sphingosine 1-phosphate is a signalling lipid known to be involved in calcium homeostasis [42–44]. *SIF2* encodes the WD40 repeat-containing subunit of Set3C histone deacetylase (HDAC) complex [45]. The Set3C binds histone H3 dimethylated at lysine 4 to mediate deacetylation of histones in 5′-transcribed regions [46]. Interestingly, the chaperone mammalian relative of DnaJ (Mrj) decreases the occupancy of nuclear factor of activated T cells (NFAT), the mammalian functional counterpart of yeast Crz1, on the tumor necrosis factor-alpha promoter in cardiomyocytes in an HDAC-dependent manner [47]. A similar role might exist for Set3C on the regulation of Crz1 in gene transcription in response to calcium stress.

**Fig. 5:**
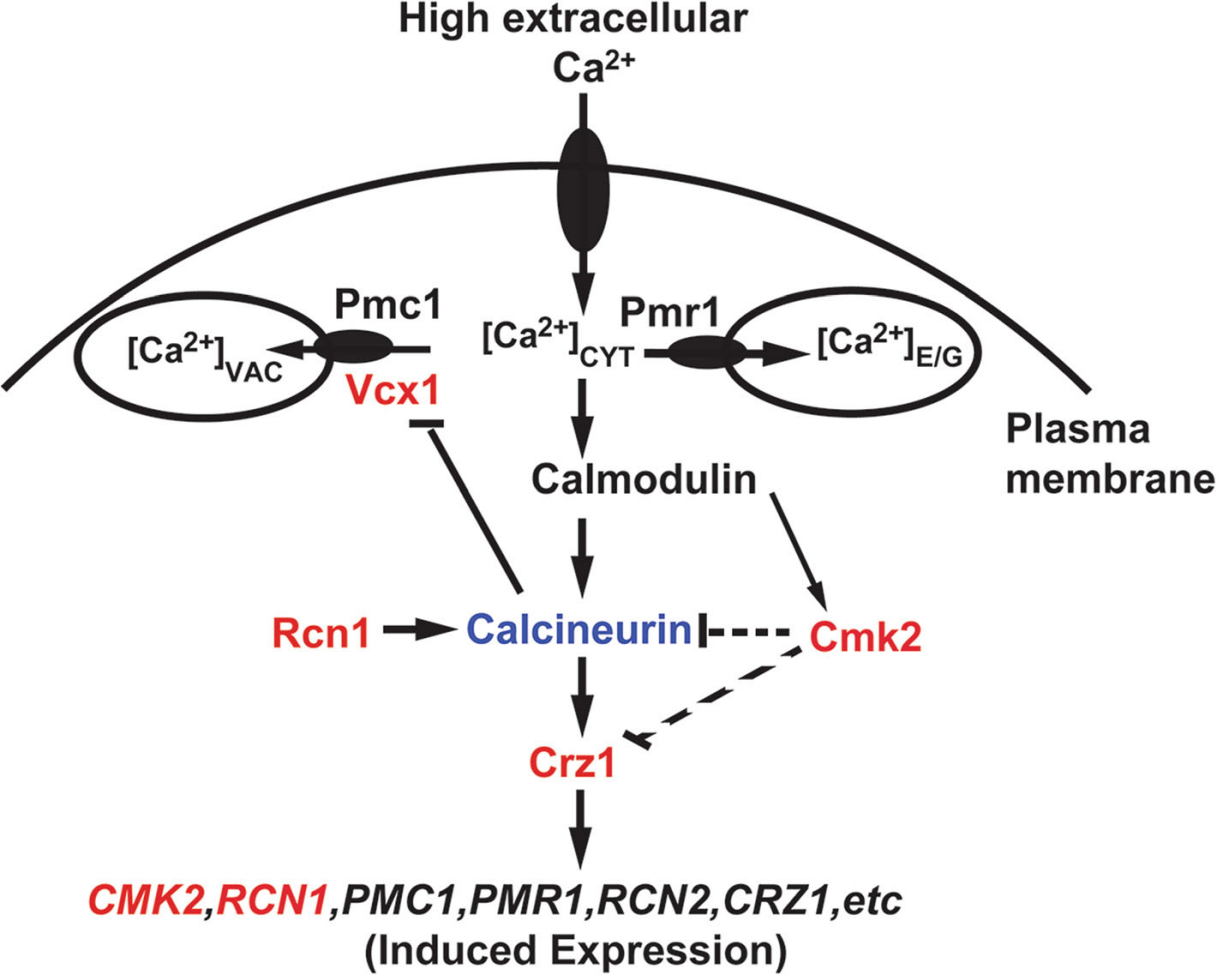
Mode of Cmk2 function in the feedback regulation of calcium/calcineurin signaling pathway. In response to high levels of extracellular calcium, Ca^2+^ enters the cell through unknown pathways, and both calcineurin and Crz1 are activated to induce expression of target genes including *CMK2*, *RCN1*, *PMR1* and *PMC1* [50]. Arrows indicate activation, and T bars represent inhibition. A dashed line means that more data is needed to confirm

*CMK2* encodes a calmodulin-dependent protein kinase, and expression of *CMK2* is induced by the calcium/calcineurin signalling pathway [48, 49]. Yeast cells lacking *CMK2* are tolerant to high levels of external calcium, while cells lacking the *CRZ1* are calcium-sensitive. Therefore, Cmk2 and Crz1 have opposite functions in the calcium sensitivity of yeast cells. In addition, we show here that deletion of *CMK2* elevates the calcium/calcineurin signalling and increases the expression level of *PMR1* and *PMC1* in response to calcium stress (Fig. 3). The effects of Cmk2 on calcium sensitivity and calcium/calcineurin signalling are dependent on its catalytical activity (Fig. 4). Taken together, these data suggest that Cmk2 functions as a negative feedback controller for the calcium/calcineurin signalling pathway, which is mediated by Crz1 through calcineurin in response to high levels of extracellular calcium stress (Fig. 5). How Cmk2 regulates the calcium signalling pathway remains to be determined. A plausible possibility would be that Cmk2 directly phosphorylates and negatively regulates calcineurin or Crz1 (Fig. 5). In contrast, Rcn1 is a feedback controller of calcineurin with dual effects [48, 50]. Another possibility is that Cmk2 indirectly affects Set3C, which in turn regulates the activity of Crz1.

Interestingly, we have observed that the *cmk2 crz1* double deletion mutant is more tolerant to 0.2 M CaCl_2_, albeit not to a higher level of calcium (0.4 M CaCl_2_), than the *crz1* deletion mutant (Fig. 2d). This indicates that Cmk2 has an additional function in calcium tolerance, which is independent of Crz1. However, supplementation of CsA in the medium suppresses the calcium sensitivity of both the *crz1* and *cmk2/crz1* mutants at both 0.2 M CaCl_2_ and 0.4 M CaCl_2_ (Fig. 2d). This indicates that the additional Crz1-independent function of Cmk2 is dependent on calcineurin function (Fig. 5). This additional function could be mediated by the Vcx1, whose activity can be inhibited by calcineurin [34]. Nevertheless, the direct phosphorylation and thereby regulation of Vcx1 activity by Cmk2 could not be excluded. This might also explain what we have observed that higher promoter activities of CDRE-*lac*Z and PMR1-*lac*Z in the *cmk2* mutant than those in the wild type strain under normal growth conditions (Fig. 3), when the calcium/calcineurin signaling pathway is not activated.

## Additional file

Additional file 1: **Figure S1.** Amino acid sequence comparison between ScCMK2, SpCMK2 and ScRCK2. (PDF 88 kb)

## Abbreviations

CaMK: Calmodulin-dependent protein kinase
CDRE: Calcineurin dependent response element
CsA: Cyclosporine A
ER: Endoplasmic reticulum
NFAT: Nuclear factor of activated T cells
PCR: Polymerase chain reaction
YPD: Yeast peptone dextron
