# Supplementary material for: The protein kinase Cmk2 negatively regulates the calcium/calcineurin signalling pathway and expression of calcium pump genes PMR1 and PMC1 in budding yeast
Source: Cell Commun Signal. 2019 Jan 21;17:7. doi: 10.1186/s12964-019-0320-z (PMC6341702; doi:10.1186/s12964-019-0320-z)
Supplement: Supplementary file 1 — Figure S1. Amino acid sequence comparison between ScCMK2, SpCMK2 and ScRCK2. (PDF 88 kb) [file 12964_2019_320_MOESM1_ESM.pdf]

```
ScCMK2    (1) ----------------------------------------------------------------------------------------------------
SpCMK2    (1) ----------------------------------------------------------------------------------------------MSILAG
ScRCK2    (1) MLKIKALFSKKKPDQADLSQESKKPFKGKTRSSGTNNKDVSQITSSPKKSFQDKNIVQYPSVVADDHHMKSLTDELVTTIDSDSSPSDNITTENVETVTS

ScCMK2    (1) -------------MPKESEVINSEFHVDVQDPERLNGHPVAKFINKLSGQPES---YVNRTNYIFGRTLGAGSFGVVRQARKLSTNED---------VAI
SpCMK2    (7) FKNLLKHSKSSKGRSNASKSVDVSVNRDVAAYTELAAKNVNAGGDEEIRVAN----YPGLEKYQLIENLGDGAFSQVYKAYSIDRKEH---------VAV
ScRCK2  (101) VPAIDVHESSEGQLSSDPLISDESLSEQSEIISDIQDDSTDDDNMEDEIPEKSFLEQKELIGYKLINKIGEGAFSKVFRAIPAKNSSNEFLTKNYKAVAI

ScCMK2   (76) KILLKKALQG---------------NNVQLQMLYEELSILQKLSHP--NIVSFKDWFESKDKFYIVTQLATGGELFDRILSRGKFTEVDAVEIIVQILGA
SpCMK2   (94) KVIRKYEMN-----------------KKQRQGVFKEVNIMRRVKH--KNVVNLFDFVETEDFYHLVMELAEGGELFHQIVNFTYFSENLARHIIIQVAEA
ScRCK2  (201) KVIKKADLSSINGDHRKKDKGKDSTKTSSRDQVLKEVALHKTVSAGCSQIVAFIDFQETDSYYYIIQELLTGGEIFGEIVRLTYFSEDLSRHVIKQLALA

ScCMK2  (159) VEYMHSKN-VVHRDLKPE-------NVLYVDK-----------------SENS-------PLVIADFGIAKQLKGEEDLIYKAAGSLGYVAPEVLTQDGH
SpCMK2  (175) VKHLHDVCGIVHRDIKPENLLFQPIEYLPSQNYTPPSLEP--NKLDEGMFLEGIGAGGIGRILIADFGFSKVVWNSKTATP--CGTVGYAAPEIVNDELY
ScRCK2  (301) VKHMHSLG-VVHRDIKPENLLFEPIEFTRSIKPKLRKSDDPQTKADEGIFTPGVGGGGIGIVKLADFGLSKQIFSKNTKTP--CGTVGYTAPEVVKDEHY

ScCMK2  (227) GKPCDIWSIGVITYTLLCGYSPFIAESVEGFMEECTASRYPVTFHMPYWDNISIDVKRFILKALRLNPADRPTATELLDDPWITSKRVETSN----ILPD
SpCMK2  (271) SKNVDMWAMGCVLHTMLCGFPPFFDENIKDLASKVVNG--EFEFLSPWWDDISDSAKDLITHLLTVDPRERYDIHQFFQHPWIKGESKMP----------
ScRCK2  (398) SMKVDMWGIGCVLYTMLCGFPPFYDEKIDTLTEKISRG--EYTFLKPWWDEISAGAKNAVAKLLELEPSKRYDIDQFLDDPWLNTFDCLP----------

ScCMK2  (323) VKKGFSLRKKLRDAIEIVKLN---------------NRIKRLRNMYSLGDDG-DNDIEENSLNESLLDGVTHSLDDLRLQSQKKGGE----LTEEQMKLK
SpCMK2  (359) --ENFTYKPKLHGTPGGPKLSLPRSLVSKGEIDIPTTPIKSATHPLLSSYSEPKTPGVSSVHEAMGVAYDIRRLNHLGFSPEQLSKKSMNTGSIKELILD
ScRCK2  (486) --KEGESSQKKAGTSE----R--R------------HPHKKQFQLFQRDSSLLFSPAAVAMRDAFDIGNAVKRTEEDRMGTRGGLGSLAEDEELEDSYSG

ScCMK2  (403) SALTKDAFVQIVKAATKNKHKVLAGE--EEDDSKKTLHDDRESKSED-----------
SpCMK2  (457) EETTTDDDDYIISSFPLNDTLGSEGKDPFSLNLKESSLYSRRSAKRVN----------
ScRCK2  (566) AQGDEQLEQNMFQLTLDTSTILQRRKKVQENDVGPTIPISATIRE-------------
```

Figure S1. Amino acid sequence comparison between ScCMK2, SpCMK2 and ScRCK2. Identical amino acid residues are indicated in yellow in color, and similar amino acid residues are indicated in light blue and green. The lysine residues (K) critical to their catalytical activity are indicated in purple. *Saccharomyces cerevisiae* ScCMK2 (GenBank Accession No: NP_014626.1), *Schizosaccharomyces pombe* SpCMK2 (Accession No: NP_594436.1), *S. cerevisiae* ScRCK2 (Accession No: NP_013349.1).
